# Th2 cells inhibit growth of colon and pancreas cancers by promoting anti-tumorigenic responses from macrophages and eosinophils

**DOI:** 10.1038/s41416-022-02056-2

**Published:** 2022-11-14

**Authors:** Damian Jacenik, Ioannis Karagiannidis, Ellen J. Beswick

**Affiliations:** 1 Department of Cytobiochemistry, Faculty of Biology and Environmental Protection, University of Lodz, Lodz, Poland; 2 Division of Gastroenterology, Hepatology and Nutrition, Department of Internal Medicine, University of Utah, Utah Salt Lake City, USA

**Keywords:** Translational research, Cancer immunotherapy, Preclinical research

## Abstract

**Background:**

Immunotherapy of gastrointestinal cancers is challenging; however, several lines of evidence suggest that adoptive transfer of stimulated or modified immune cells support not only protective role of immune cells in tumor microenvironment, but actively participate in the elimination of cancer cells.

**Methods:**

In vivo studies employing cancer cell-derived allograft murine models of gastrointestinal cancers were performed. The effects of T helper (Th) 2 cells on gastrointestinal cancers growth and tumor microenvironment composition using adoptive transfer of Th2 cells, interleukin (IL)-5 treatment, and immunofluorescence, multiplex and real-time PCR were explored.

**Results:**

Here, we show that Th2 cells play an essential role in the inhibition of colon and pancreas cancers progression. In murine models of gastrointestinal tumors using adoptive transfer of Th2 cells, we identify that Th2 cells are responsible for generation of apoptotic factors and affect macrophage as well as eosinophil recruitment into tumors where they produce cytotoxic factors. Moreover, we found that Th2 cells lead to IL-5 hypersecretion, which links the anti-tumorigenic function of Th2 cells and eosinophils. Importantly, we noted that recombinant IL-5 administration is also related with inhibition of gastrointestinal tumor growth. Finally, using an in vitro approach, we documented that both Th2 cells and eosinophils are directly responsible for gastrointestinal cancer cell killing.

**Conclusions:**

These data demonstrate the significance of Th2 cells, eosinophils and IL-5 in the inhibition of gastrointestinal tumor growth, and pointed toward tumor microenvironment reprogramming as a Th2 cell-mediated anti-tumorigenic mechanism of action.

## Background

The bidirectional communication between cancer cells and immune cells affects the development and progression of cancers. It has been well-documented that the level of immune cell infiltration and/or immune cell composition in tumor microenvironment (TME) correspond to the progression of cancers and survival time of patients with cancer [1–3]. Immunotherapy is a promising treatment approach, but unfortunately immune-based therapies have limited effectiveness in patients with some cancers, and only a subset of gastrointestinal tumors respond to immunotherapy. For instance, Le et al. found that patients with progressive and metastatic carcinoma with defective, but not proficient mismatch repair tumors, are better candidates for immunotherapy based upon immune checkpoint inhibition [4]. On the other hand, in patients with pancreatic ductal adenocarcinoma adoptive immunotherapy using stimulated cytotoxic T cells in combination with gemcitabine seems to be related with metastasis prevention. It should be mentioned, that activating T cells has been a mainstay of studying anti-tumorigenic immunity. To note, liver metastasis was found in 33% and local recurrence occurred in 19% of patients with pancreas cancer treated with combination of cytotoxic T cells and gemcitabine [5]. In fact, a challenge in the field of tumor immunotherapy is the immunosuppressive microenvironment surrounding tumor cells. Thus, novel approaches to reprogramming immune responses in TME may be a key to unlocking an anti-tumorigenic immunity.

In addition to adoptive cell therapy or checkpoint blockade immunotherapies, T cell phenotype may be a critical component of the TME. T helper (Th) 2 cells are most well-known for their role in allergic responses, but have not been well studied in cancers [6]. Studies to date with type II immune responses have shown protective function in some cancers, but the mechanisms associated with the protective response are not well understood, and the overall published data remains contradictory. Particularly, the impact of Th2 cells and associated cytokines in cancer are not well studied mechanistically. The information available on these cytokines in tumors has been mixed and mostly descriptive [7]. For instance, interleukin (IL)-5 has been shown in some studies to support anti-tumorigenic responses, perhaps through eosinophils [8, 9]. Although the potential of type II immune responses has been somewhat overlooked, there is evidence that some components may support anti-tumorigenic immunity. IL-5 is known to promote eosinophil migration and function and although not well studied in tumor settings, eosinophils are thought to have cytotoxic effects on cancer cells [10]. These effects may be through secreted cytotoxic factors, such as granzyme B (GZMB) or major basic protein (MBP) [11]. In a previous study, where mice with IL-5 depletion in a model of sarcoma were employed, the protective role of both IL-5 and eosinophils was observed [9]. Moreover, there are some studies suggesting that eosinophils support tumoricidal action in in vitro and in vivo models of colon cancer, thus suggesting these responses should be further investigated [12–14].

We postulate that Th2 cells may be crucial regulators of colon and pancreas cancer progression by affecting immune cell composition and type II immune responses. In this study, we evaluate the therapeutic potential of Th2 cells on the progression of gastrointestinal cancers. In murine allograft models of colon and pancreatic cancers, adoptive transfer of Th2 cells into tumor-bearing mice led to substantially reduced tumor growth. Th2 cells were shown to induce cytotoxicity by supporting innate immune cell responses, including eosinophils and macrophages. Furthermore, we show that IL-5 provides protection against tumor growth by recruiting and activating eosinophils. Overall, this study is the first to show a directly protective effect by Th2 cells and type II immune responses that could be potential therapeutic targets and could be utilized for gastrointestinal cancers.

## Methods

### Mice

C57BL/6 wild type and B6.129S7-*Rag1*^*tm1Mom*^/*J* (*Rag1*^−/−^) mice were obtained from the Jackson Laboratory (Bar Harbor, MA, USA) and bred in-house. The animals were housed in the Comparative Medicine Center, University of Utah Health, UT, USA at constant temperature (22–24 °C), relative humidity ~55% and maintained under 12 h light/dark cycle with access to standard chow pellets and tap water *ad libitum*. The research has been approved by the University of Utah Institutional Animal Care and Use Committee. Every effort was taken to minimize animal suffering and to reduce the number of animals used.

### Isolation and polarization of naive CD4^+^ T cells

Spleens were harvested from C57BL/6 wild type mice and naive CD4^+^ T cells were isolated by magnetic separation using Naive CD4^+^ T-Cell Isolation Kit (cat. 130-104-453, Miltenyi Biotec., Auburn, CA, USA). The naive CD4^+^ T-cell polarization was performed according to the manufacturer’s instructions using the CytoBox Th2 (cat. 130-107-760, Miltenyi Biotec., Auburn, CA, USA). The isolated naive CD4^+^ T cells were resuspended in TexMACS^™^ media (cat. 130-097-196, Miltenyi Biotec., Auburn, CA, USA), supplemented with 10% heat-inactivated fetal bovine serum (ThermoFisher Scientific, Waltham, MA, USA), 1% penicillin/streptomycin (Corning, Tewksbury, MA, USA), 50 U/ml mouse IL-2 (CytoBox Th2, Miltenyi Biotec., Auburn, CA, USA), 200 U/ml mouse IL-4 (CytoBox Th2, Miltenyi Biotec., Auburn, CA, USA) and 10 μl/ml anti-IFN-γ antibody (CytoBox Th2, Miltenyi Biotec., Auburn, CA, USA). 2 × 10^5^ of naive CD4^+^ T cells were transferred to 96-well round-bottom plate, activated using T-cell activation beads coupled with anti-CD3 and anti-CD28 antibodies (cat. 11456D, ThermoFisher Scientific, Waltham, MA, USA) and incubated at 37 °C and 5% CO_2_. After 6 days of differentiation of naive CD4^+^ T cells, Th2 cells were collected and used for in vitro or in vivo analyses.

### Isolation of eosinophils

Eosinophils were isolated from spleens of C57BL/6 wild type mice by magnetic separation using Anti-Siglec-F MicroBeads (cat. 130-118-513, Miltenyi Biotec., Auburn, CA, USA) according to manufacturerʼs protocol. After separation, eosinophils in RPMI media (Corning, Tewksbury, MA, USA) supplemented with 10% heat-inactivated fetal bovine serum (ThermoFisher Scientific, Waltham, MA, USA), 1% penicillin/streptomycin (Corning, Tewksbury, MA, USA) and 1% l-glutamine (ThermoFisher Scientific, Waltham, MA, USA) were resuspended and used for in vitro studies.

### Murine allograft models and treatments

BRAF (BRAF^V600EΔTRZI^) cells were derived from a mouse tumor developed from organoids kindly shared by Dr. Daniel Worthley from the South Australian Medical and Health Institute, Australia [15]. The tumor was dissociated using the gentleMACS™ instrument (Miltenyi Biotec., Auburn, CA, USA) and cultured in RPMI media (Corning, Tewksbury, MA, USA) supplemented with 10% heat-inactivated fetal bovine serum (ThermoFisher Scientific, Waltham, MA, USA), 1% penicillin/streptomycin (Corning, Tewksbury, MA, USA) and 1% l-glutamine (ThermoFisher Scientific, Waltham, MA, USA). After several passages, the resulting cell line was utilized to induce tumors for this study. PK5L1940 cells were provided by Dr. Michael Gough from the Earle A. Chiles Research Institute, Portland, OR, USA [16]. Cells were cultured in RPMI media (Corning, Tewksbury, MA, USA) supplemented with 10% heat-inactivated fetal bovine serum (ThermoFisher Scientific, Waltham, MA, USA), 1% penicillin/streptomycin (Corning, Tewksbury, MA, USA) and 1% l-glutamine (ThermoFisher Scientific, Waltham, MA, USA). In all studies, cells testing negative for mycoplasma were used. BRAF cells (2 × 10^6^) or PK5L1940 cells (1 × 10^6^) resuspend in PBS and mixed with Matrigel^®^ (Corning, Tewksbury, MA, USA) were injected into the flank of 6–10-week-old male or female randomized *Rag1*^−/−^ mice, investigators were not blinded. Ten animals per cell line were used in tumor experiments. Tumors were manually measured using caliper starting from day 1. Some mice with BRAF and PK5L1940 cell-derived allografts were administered intratumorally with 5 × 10^5^ of Th2 cells or 200 ng of IL-5 (cat. 200–20, Shenandoah Biotechnology, Inc., Warwick, PA, USA) resuspended in PBS. Tumor size was calculated according to the following formula: tumor size = (length × length × width)/2. Mice reached the endpoint when tumor volumes were approximately 2000 mm^3^.

### Immunohistochemistry analysis

Tumors were fixed in 4% paraformaldehyde for up to 24 h, incubated in 15% and then 30% sucrose-PBS solutions for up to 12 h, each. Tumor pieces were embedded in the Tissue-Plus™ O.C.T. Compound Tissue-Plus™ (cat. 23-730-571, ThermoFisher Scientific, Waltham, MA, USA). Sections (5 μm) were blocked with 2% normal serum and incubated with commercially available antibodies against F4/80-PE (cat. 11-4801-82), GATA3-PE (cat. 46-9966-41), MBP (cat. MA1-24990), MPO (cat. PA5-16672), NOS2-APC780 (cat. 47-5920-82), SIGLEC-F-PE (cat. 552125). The above-mentioned antibodies conjugated and unconjugated with fluorochrome were used at 1:200 dilution and were purchased from ThermoFisher Scientific (Waltham, MA, USA) or BD Bioscience (San Diego, CA, USA). The sections where antibodies conjugated with fluorochrome were incubated for 1 h at room temperature. The sections where unconjugated with fluorochrome antibodies were used were incubated overnight at 4°C. Next, the sections were washed and incubated with donkey anti-rat secondary antibodies (cat. A-21209, ThermoFisher Scientific, Waltham, MA, USA) for 1 h. Subsequently, the sections were washed with PBS and mounted in SlowFade™ Gold Antifade Mountant with DAPI (cat. S36938, ThermoFisher Scientific, Waltham, MA, USA). The sections were analyzed using EVOS™ M7000 Imaging System (ThermoFisher Scientific, Waltham, MA, USA) featuring 10x and 20x objectives.

### Tumor-killing assay and flow cytometry analysis

BRAF and PK5L1940 cells were plated and Th2 cells or eosinophils in a ratio 1:2 were added. Cells were incubated for up to 24 h and co-cultures stained with CellEvent™ Caspase-3/7 Green Detection Reagent (cat. C10423, ThermoFisher Scientific, Waltham, MA, USA). Supernatants from co-cultures were collected for multiplex analysis. Flow cytometry analysis was performed using an Attune™ NxT Flow Cytometer (ThermoFisher Scientific, Waltham, MA, USA) and analyzed with Attune™ NxT Software (ThermoFisher Scientific, Waltham, MA, USA).

### RNA extraction, reverse transcription and real-time PCR

Tumor pieces were homogenized in TRIzol™ reagent (cat. 15596026, ThermoFisher Scientific, Waltham, MA, USA) and RNA extraction was performed according to the manufacturer’s instructions. The quality and quantity of RNA were measured with a NanoDrop™ Lite Spectrophotometer (ThermoFisher Scientific, Waltham, MA, USA). Total RNA (100 ng/µl) was reverse transcribed using High-Capacity cDNA Reverse Transcription Kit (cat. 4368814, ThermoFisher Scientific, Waltham, MA, USA) with the following PCR settings: 25 °C for 10 min, 37 °C for 120 min and 85 °C for 5 min. Quantitation of mRNA was performed using real-time PCR with validated FAM dye-labelled TaqMan^®^ probes (Applied Biosystems, Foster City, CA, USA) for *Actb*—Mm02619580_g1, *Adgre1*—Mm00802529_m1, *Fas*—Mm01204974_m1, *Fasl*—Mm00438864_m1, *Gsr*—Mm00439154_m1, *Gzmb*—Mm00442837_m1, *Mbp*—Mm01266402_m1, *Mpo*—Mm01298424_m1, *Nos2*—Mm00440502_m1, *Prf1*—Mm00812512_m1, *Siglef*—Mm00523987_m1. The reaction mixture consisted of cDNA, TaqMan^®^ Fast Advanced Master Mix (Applied Biosystems, Foster City, CA, USA), TaqMan^®^ Assays, and RNase-free water in a total volume of 10 μl. Cycle parameters for TaqMan^®^ assays were as follows: initial denaturation at 95 °C for 3 min, followed by 40 cycles of sequential incubations at 95 °C for 15 s and 60 °C for 1 min. Results were normalized to the expression of housekeeping gene, i.e., *Actb*. All experiments were performed at least as duplicates on QuantStudio™ 5 Real-Time PCR System (ThermoFisher Scientific, Waltham, MA, USA). The endpoint used in real-time PCR quantification—CT—was defined as the PCR cycle number that crossed the signal threshold. Quantification of gene expression was performed using the comparative CT method (Sequence Detector User Bulletin 2; Applied Biosystems) and reported as the fold-change relative to the mRNA of the mouse housekeeping gene.

### Multiplex analysis

BRAF and PK5L1940 cell-derived allograft tumors were divided into 8 mg pieces (± 0.5 mg) and incubated in RPMI media (Corning, Tewksbury, MA, USA) supplemented with 10% heat-inactivated fetal bovine serum (ThermoFisher Scientific, Waltham, MA, USA), 1% penicillin/streptomycin (Corning, Tewksbury, MA, USA) and 1% l-glutamine (ThermoFisher Scientific, Waltham, MA, USA) up to 18 h. Tumor culture supernatants were analyzed for cytokine and chemokine levels by multiplex arrays (MilliporeSigma, Burlington, MA, USA) and Luminex^®^ in accordance with the manufacturer’s instructions. Mouse cytokine panel 1 and a custom panel consisting of GZMB and FAS were used for this study (MilliporeSigma, Burlington, MA, USA).

### Statistical analyses

Statistical analysis was performed using GraphPad Prism 5.0 (GraphPad Software Inc., San Diego, CA, USA). Results are presented as means ± standard error of mean (SEM). Non-parametric Mann–Whitney *U*-test and two-way ANOVA followed by Bonferroni’s multiple comparison post hoc test were used for comparison of studied groups. *P*-values < 0.05 was considered statistically significant.

## Results

### Th2 cells decrease the growth of colon and pancreas cancers

**Fig. 1:**
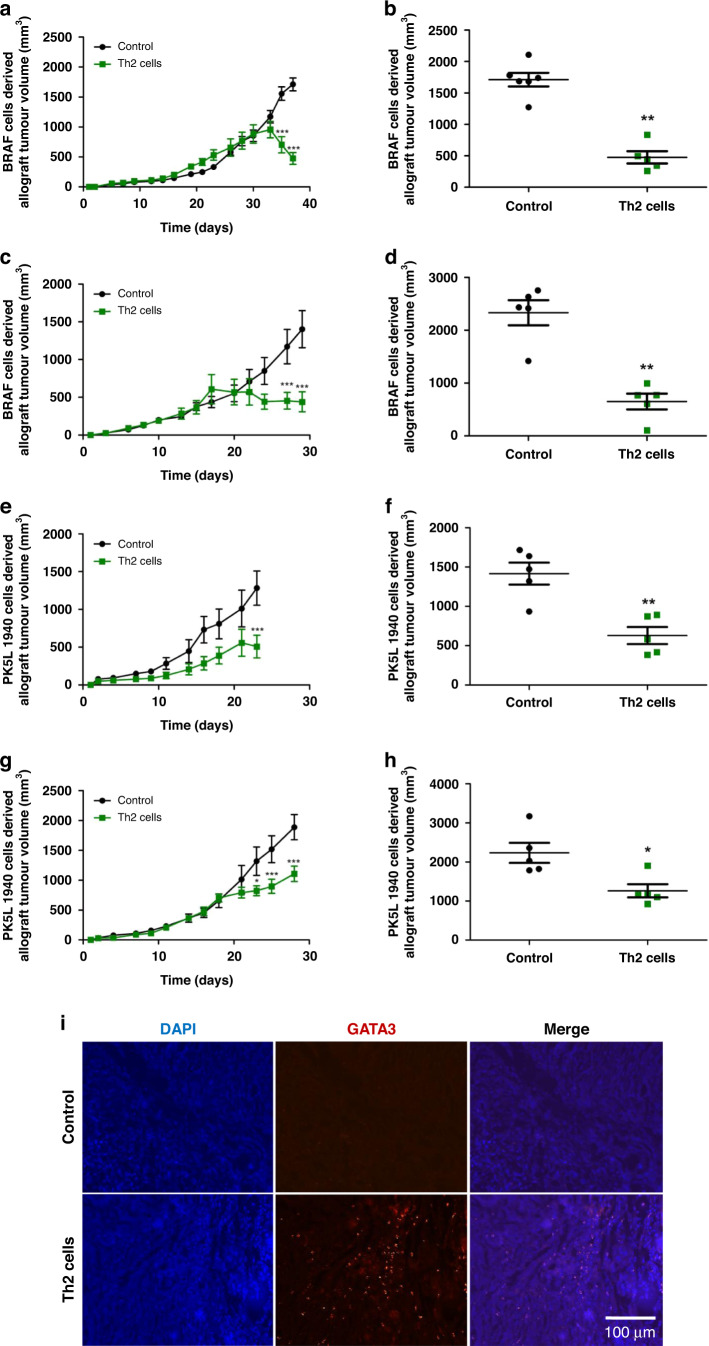
Th2 cell administration inhibits growth of gastrointestinal cancers. The growth profile (**a**, **c**, **e** **g**) and tumor volume (**b**, **d**, **f**, **h**) of BRAF (**a**, **c**) and P5KL1940 cells (**e**, **g**) -derived allografts in untreated mice and mice with weekly (**a**, **c**, **e**) and one time (**g**) injection of Th2 cells showing decreased tumor growth. Representative images of immunohistochemistry staining of GATA3 (**i**) in PK5L1940 cell-derived allograft tumors obtained from untreated *Rag1*^−/−^ mice (control) and *Rag1*^−/−^ mice with one injection of Th2 cells (Th2 cells). Data are presented as means ± SEM; ^*^P <  0.05, ^***^P <  0.001 vs. control. Scale bars, 100 µm.

To investigate the role of immune responses mediated by Th2 cells in the progression of gastrointestinal tumors, BRAF and P5K1940 cell-derived allograft tumor models were employed. To note, PK5L1940 pancreatic cancer cells are characterized by *Kras* mutation, while BRAF (BRAF^V600EΔTRZI^) colorectal cancer cells characterized by *Braf* mutation were obtained from organoids and subcutaneous inoculation of these cells in animals that led to the development of serrated colon tumors [15, 16]. As was shown in Fig. 1a–h, we observed markedly suppressed growth of BRAF and PK5L1940 cell-derived allograft tumors in male and female mice treated with Th2 cells when compared to control animals. It should be noted that after naive T cells polarization, Th2 cells were expressing high levels of GATA3 along with enhanced production of IL-5, lower levels of IL-4 and IL-13, as well as very little level of interferon-γ (IFN-γ) (Supplementary Fig. S1). Interestingly, we found that in the PK5L1940 cell-derived allograft tumors, one administration of Th2 cells was able to significantly decrease tumor growth by ~50%, but not regress tumors as weekly injections of Th2 cells did (Fig. 1g, h). Moreover, in the animals that received one treatment, Th2 cells were detected in PK5L1940 cell-derived tumors on the last day of the experiment, which was evaluated by immunofluorescence staining of GATA3, suggesting longevity of these cells in the TME (Fig. 1i). Overall, we found that Th2 cell administration suppresses tumor growth so these data suggest a protective effect of Th2 cell in gastrointestinal tumors.

### Th2 cells increase eosinophil and macrophage influx in colon and pancreas cancers

**Fig. 2:**
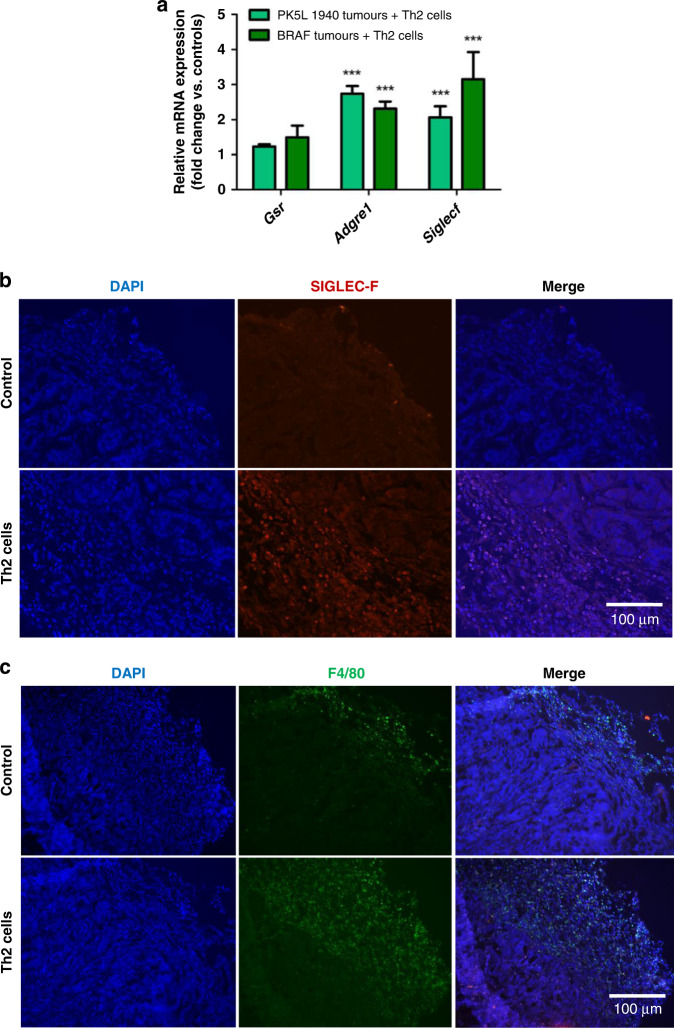
Th2 cell administration induces eosinophil and macrophage accumulation in gastrointestinal cancers. The expression of *Gsr*, *Adgre1* and *Siglecf* (**a**) at the mRNA level in PK5L1940 and BRAF cell-derived allograft tumors obtained from *Rag1*^−/−^ mice injected with Th2 cells in relation to untreated *Rag1*^−/−^ mice are increased. Representative images of immunohistochemistry staining showing that SIGLEC-F (**b**) and F4/80 (**c**) levels in PK5L1940 cell-derived allograft tumors obtained from untreated *Rag1*^−/−^ mice (control) and *Rag1*^−/−^ mice injected with Th2 cells are increased. Data are presented as means ± SEM; ^***^P <  0.001 vs. control. Scale bars, 100 µm.

In order to understand the impact of Th2 cells on the TME modulation, we examined the innate cell immune profile of the BRAF and PK5L1940 cell-derived allograft tumors. Real-time PCR analysis indicated increased expression of several immune cell markers in BRAF and PK5L1940 cell-derived allograft tumors obtained from animals treated with Th2 cells when compared to control animals. We found that the expression of *Siglecf*, a marker for eosinophils, was increased 2-fold in PK5L1940 and 3-fold in BRAF tumors, after Th2 administration when compared to control animals (Fig. 2a). The expression of *Adgre1* (F4/80, a macrophage marker) was increased by up to 3-fold in both tumor types, while *Gsr*, a marker of neutrophils and other granulocytes was not significantly increased in either tumor type. The influx of eosinophil and macrophage were further examined by immunofluorescence where strong SIGLEC-F staining in PK5L1940 cell-derived allograft tumors obtained from animals treated with Th2 cells when compared to control animals was observed (Fig. 2b). Our analysis revealed substantially increased F4/80 staining in tumor cross-sections obtained from animals treated with Th2 cells in relation to control animals (Fig. 2c). These results suggest a major influx of eosinophils as Th2 cells are known to support in other diseases; however, a novel finding is that there is an increase in F4/80 expressing macrophages.

### Th2 cells increase innate immune cell activity

**Fig. 3:**
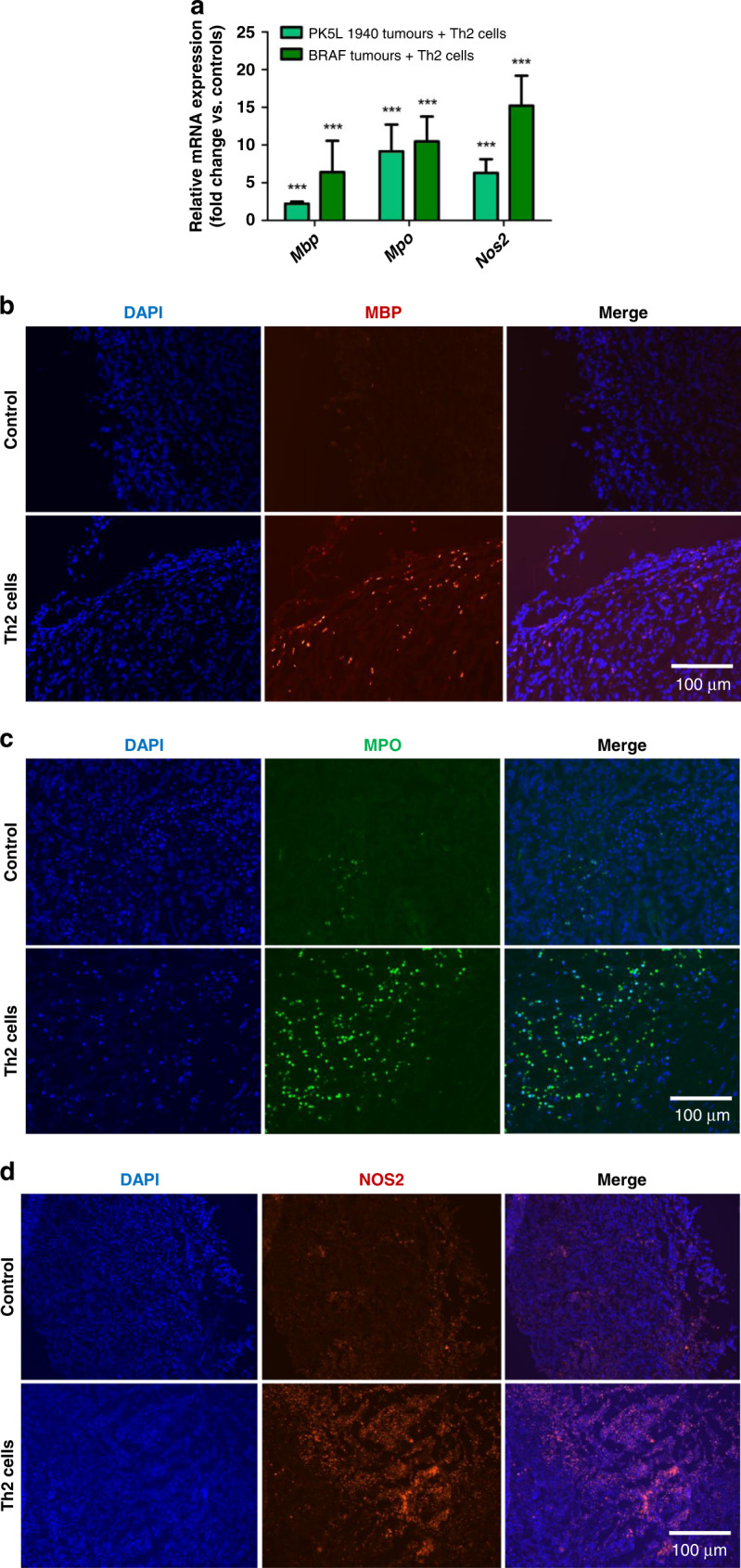
Th2 cell administration enhances innate immune cell activity in gastrointestinal cancers. The expression of *Mbp*, *Mpo* and *Nos2* (**a**) at the mRNA level in PK5L1940 and BRAF cell-derived allograft tumors obtained from *Rag1*^−/−^ mice injected with Th2 cells in relation to untreated *Rag1*^−/−^ mice are increased. Representative images of immunohistochemistry staining showing that MBP (**b**), MPO (**c**) and NOS2 (**d**) levels in PK5L1940 cell-derived allograft tumors obtained from untreated *Rag1*^−/−^ mice (control) and *Rag1*^−/−^ mice injected with Th2 cells are increased. Data are presented as means ± SEM; ^***^P <  0.001 vs. control. Scale bars, 100 µm.

Since we found decreased growth of BRAF and PK5L1940 tumors and increased influx of eosinophils and macrophages into BRAF and PK5L1940 tumors after administration of Th2 cells, we next set out to examine the activity of these cells in BRAF and PK5L1940 tumor models. The gene expression of the eosinophil factor *Mbp* was found to be increased by 2-fold in PK5L1940 and 6-fold in BRAF tumors (Fig. 3a). Higher protein levels of MBP was also detected by immunofluorescence in tumors obtained from animals treated with Th2 cells in relation to tumors from control animals (Fig. 3b). Furthermore, up to 10-fold increased expression of *Mpo* at the mRNA and protein levels in both tumor types obtained from animals treated with Th2 cells in relation to control animals (Fig. 3a, c). MPO is most well-known to be produced by neutrophils, but may also be produced by macrophages and may have the ability to induce apoptosis in tumor cells [17, 18]. As another marker of macrophage activity, *Nos2* was examined. P5KL1940 tumors obtained from animals treated with Th2 cells were characterized by a 5-fold increase in *Nos2* gene expression compared to control tumors, while BRAF tumors taken from animals treated with Th2 cells had up to a 15-fold increase the expression of *Nos2* compared to control tumors (Fig. 3a). Along with the above-mentioned data, a higher level of NOS2 in PK5L1940 tumors obtained from animals treated with Th2 cells compared to control tumors was confirmed using immunofluorescence analysis (Fig. 3d). These results suggest that not only innate immune cells are increased in tumors treated with Th2 cells, but also enhanced activity of these cells was noted, suggesting potential mechanism of the anti-tumorigenic action of Th2 cells.

### Th2 cells and eosinophils induce the expression of cytotoxic/apoptotic factors

**Fig. 4:**
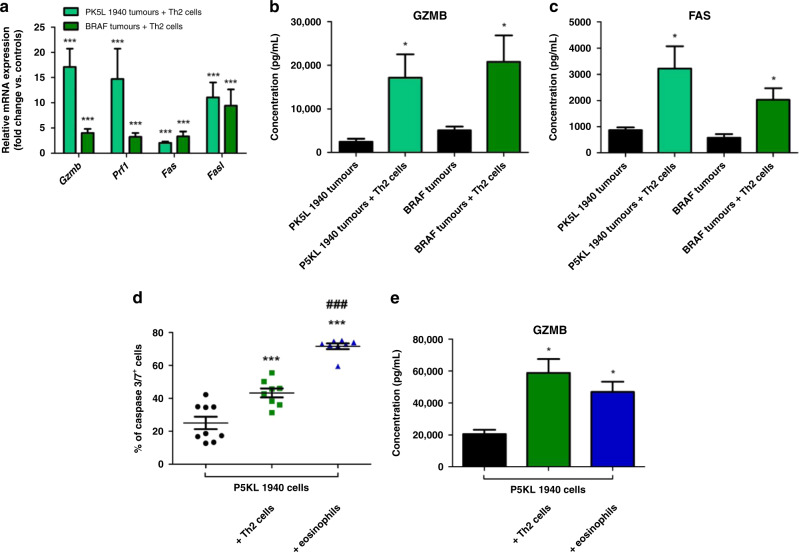
Th2 cells and eosinophils promote expression and secretion of anti-tumorigenic factors in gastrointestinal cancers. The expression of *Gzmb*, *Prf1*, *Fas* and *Fasl* (**a**) at the mRNA level in PK5L1940 and BRAF cell-derived allograft tumors obtained from *Rag1*^−/−^ mice injected with Th2 cells in relation to untreated *Rag1*^−/−^ mice are increased and the concentration of GZMB (**b**) and FAS (**c**) released from both type of gastrointestinal tumors obtained from *Rag1*^−/−^ mice injected with Th2 cells in relation to untreated *Rag1*^−/−^ mice are also increased. The percent of caspase 3/7^+^ PK5L1940 cells (**d**) and concentration of GZMB (**e**) in PK5L1940 cells and co-culture of PK5L1940 cells with Th2 cells or eosinophils are increased. Data are presented as means ± SEM; ^***^P <  0.001 vs. control or PK5L1940 cells; ^###^*P* <  0.001 vs. PK5L1940 cells + Th2 cells.

Th2 cells and eosinophils have been poorly investigated for their potential impact in the gastrointestinal TME. Thus, we hypothesized that Th2 cells and eosinophiles induce the production/release of mediators that affect apoptosis of cancer cells. When cytotoxic and apoptotic markers were examined in BRAF and PK5L1940 cell-derived allograft tumors by real-time PCR, an increase of *Gzmb* and *Prf1* (from 5-fold to up to 15-fold) expression in animals treated with Th2 cells compared to control animals was found, indicating an increase in tumor lytic factors (Fig. 4a). In tumors obtained from animals treated with Th2 cells, real-time PCR analysis showed a significant increase of *Fas* and *Fasl* when compared to control animals, indicating increased apoptotic factors in these tumors. We further analyzed the tumor supernatants of tumors treated with Th2 cells compared to control and found soluble GZMB and FAS to be increased (Fig. 4b, c).

It has been noted that a subset of T helper cells are capable of cytotoxicity [19, 20]. To validate this possibility, we used a tumor-killing assay where we incubated Th2 cells or eosinophils with PK5L1940 cells. We found that Th2 cells increased percent of cleaved caspase 3/7^+^ PK5L1940 cells (Fig. 4d), suggesting a novel finding where Th2 cells may have some killing effect on tumor cells. On the other hand, eosinophils isolated from mouse spleens increased percent of cleaved caspase 3/7^+^ PK5L1940 cells quite substantially revealing the ability of eosinophils to induce tumor killing (Fig. 4d). In addition, significantly higher levels of GZMB were observed in the supernatants of Th2 cells or eosinophils with PK5L1940 cells co-cultures using multiplex analysis (Fig. 4e), but soluble FAS was not detected in co-cultures as it was in tumors. These data suggests that both Th2 cells and eosinophils are characterized by tumor-killing ability, which was confirmed by this in vitro approach.

### IL-5 is increased by Th2 cell administration in tumors and decreases tumor growth

**Fig. 5:**
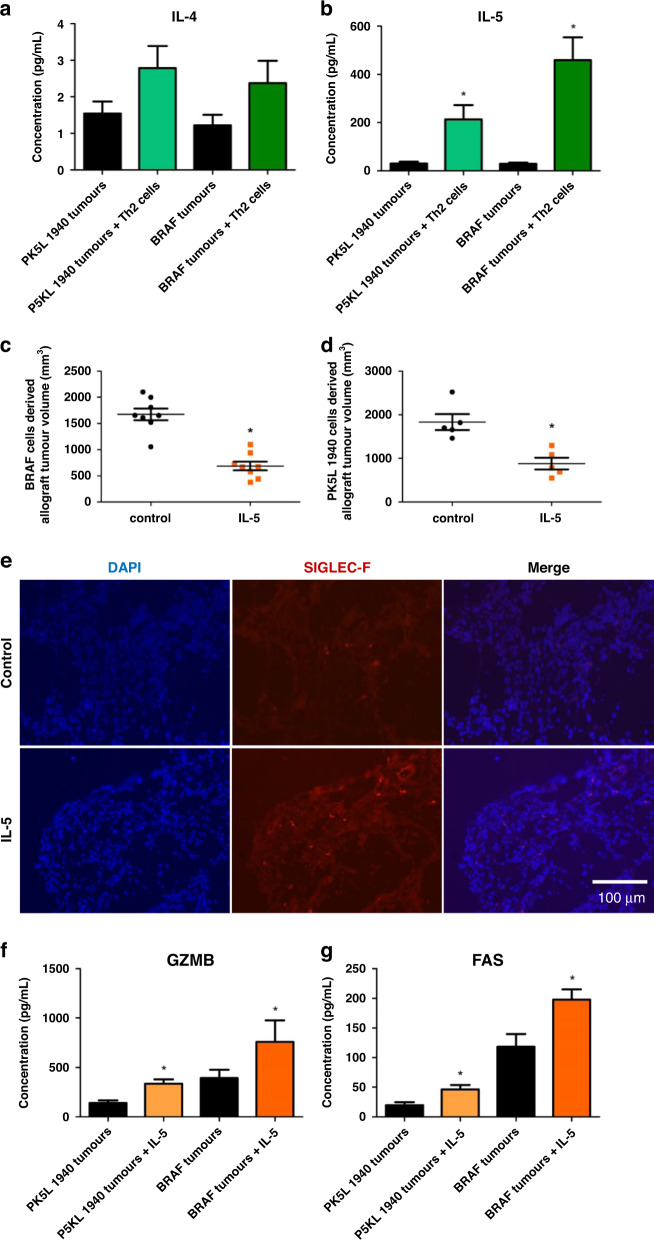
IL-5 induces anti-tumorigenic action in gastrointestinal cancers. The concentration of IL-4 (**a**) and IL-5 (**b**) released from PK5L1940 and BRAF cell-derived allograft tumors obtained from *Rag1*^−/−^ mice injected with Th2 cells in relation to untreated *Rag1*^−/−^ mice is shown by multiplex array. Tumor volume of BRAF (**c**) and PK5L1940 cell (**d**) -derived allograft tumors in untreated *Rag1*^−/−^ mice (control) and *Rag1*^−/−^ mice with weekly injection of recombinant IL-5 show decreased tumor growth with IL-5 injection. Representative images of immunohistochemistry staining of SIGLEC-F (**e**) in PK5L1940 cell-derived allograft tumors obtained from untreated *Rag1*^−/−^ mice (control) and *Rag1*^−/−^ mice injected with IL-5 show increased levels of SIGLEC-F in IL-5 treated tumors. The concentration of GZMB (**f**) and FAS (**g**) released from PK5L1940 and BRAF cell-derived allograft tumors obtained from *Rag1*^−/−^ mice injected with recombinant IL-5 in relation to untreated *Rag1*^−/−^ mice are increased in IL-5 treated mice. Data are presented as means ± SEM; ^*^P <  0.05 vs. control. Scale bars, 100 µm.

To further examine the potential of type II immune responses in promoting anti-tumorigenic immunity, we examined cytokine production in colon and pancreas tumors obtained from mice treated with Th2 cells. While we did not find a significant increase in IL-4 (Fig. 5a), which is a type II immune response cytokine, but also known to be pro-tumorigenic cytokine, we found that the type II immune cytokine IL-5 was significantly increased in mice with gastrointestinal tumors treated with Th2 cells (Fig. 5b). The type II immune cytokine IL-13 was also not detected in BRAF and PK5L1940 cell-derived allograft tumors (not shown due to lack of detection). This result led us to consider the impact of IL-5 in tumor progression. Thus, we injected recombinant IL-5 into PK5L1940 and BRAF tumor-bearing mice at biologically relevant concentrations of the amount present in tumors receiving Th2 cells. Injection of recombinant IL-5 into tumor-bearing mice, led to a ~2.5-fold decrease in tumor volume in both gastrointestinal tumor models (Fig. 5c, d). We further found an increase in eosinophil influx into tumors (Fig. 5e), but not to the extent of what was seen in tumors obtained from mice treated with Th2 cells as shown in Fig. 2a, b. Soluble GZMB and FAS were also found in tumor supernatants (Fig. 5f, g), but at lower levels compared to tumors obtained from mice treated with Th2 cells (Fig. 4b, c). Moreover, we also found an increased expression of the cytotoxic and apoptotic factors, i.e., *Gzmb* and *Fas* of up to 2–3- fold. These results suggest that IL-5 alone provides some protective effect in gastrointestinal cancers.

## Discussion

The role of type II immune responses in tumors has not been well examined. Some studies have suggested a protective role of type II immune responses in various cancers, but the majority of studies are correlative and little is known about the potential mechanisms of Th2 cells in the TME, especially in the context of its potential for therapeutic strategies. Th1 cells are traditionally thought to be the protective Th phenotype, they have also been the main focus of anti-tumor studies, while Th2 cells have been somewhat ignored. Publications to date on Th2 cells are mainly descriptive. However, there are a few studies indicating that Th2 cells may support cytotoxicity in tumors by supporting other immune cell functions [21, 22]. Furthermore, several groups have shown that Th2 cells support recruitment and function of innate immune cells [7, 23, 24]. Conversely, a few association studies suggest that Th2 cells may not improve outcome and this conviction is based on the results where a low level of Th1 cells and overall lower levels of T cell infiltration in cancers were noted. Although historically the general assumption has been that Th1 and Th2 cells are reciprocal, more recent evidence suggests T-cell plasticity and the existence of multiple phenotypes simultaneously [25]. Thus, a few studies suggest that both Th1 and Th2 cells are key immune cell types in anti-tumorigenic immunity [22].

Th2 cells are a crucial component of type II immune responses by secreting a wide spectrum of cytokines such as IL-4, IL-5, IL-9 and IL-13, which affect both tumor cells and several types of immune cells. Of note, many studies also include IL-10 in the overview of a Th2 cell response, which we know is actually indicative of type M2 macrophages and regulatory T cells [26, 27]. It was documented that IL-4 administration or overexpression is associated with reduction of tumor growth, which was estimated in in vivo studies [23]. Additionally, some studies pointed that neutralization of IL-4 using antibodies against IL-4 is directly related to the loss of anti-tumorigenic immunity and an absence of macrophages and eosinophils in tumors [28]. IL-4 and IL-5 have been shown to support eosinophil activity [9, 23]. Similar to IL-4, IL-13 is characterized by anti-tumorigenic activity and IL-13 seems to engage both macrophages and neutrophils [29–31]. Here, we found IL-5 to be the major cytokine increased upon Th2 injection. Thus, type II cytokines, particularly IL-5 may play an important role in anti-tumor immunity.

In this study, we used *Rag1*^−/−^ mice in order to investigate the impact of Th2 cells in the gastrointestinal tumors and how they regulate innate immune responses. We show that administration of naive CD4^+^ T cells polarized to Th2 cells, using IL-4, IL-2 and anti-IFN-γ antibody, not only prevented tumor growth, but reversed growth curve of colon and pancreas cell-derived allograft tumors. Interestingly, only one injection of Th2 cells led to the inhibition of tumor growth in mice, but weekly injections were more effective. This phenomenon suggests that Th2 cells generate long-lasting anti-tumorigenic immune response presumably by activation of another type/s of immune cells. To explore potential of Th2 cells in TME reprograming and tumor cell killing we employed real-time PCR and immunohistochemistry analyses. In above-mentioned analyses, we find transcriptional and proteomic evidence for eosinophil and macrophage recruitment into Th2 cell-treated tumors compared to control animals. Previous studies demonstrated that high levels of tumor-infiltrating eosinophils are positively associated with better prognosis, inversely correlated with stage and related to decreased risk of death caused by cancer progression [32, 33]. Furthermore, a low number of eosinophils is related with short overall survival and seems to be an independent prognostic factors for poor outcomes in patients with pancreatic cancer [34]. Eosinophils are a major component of the type II immune response and are capable of secreting many factors such as cytotoxic granules, lipids, growth factors, cytokines and chemokines. Mattes et al. documented functional cross-talk between Th2 cells and eosinophils for melanoma [35], where melanoma regression and clearance of lung metastases mediated by Th2 cells were dependent on eosinophil-produced chemokines and eotaxin. We found that Th2 cell administration led to MBP accumulation in gastrointestinal tumors, suggesting that Th2 cells and eosinophils cooperate to suppress the progression of both colon and pancreas cancers by releasing cytotoxic factor. To note, MBP is one of the main effectors released by eosinophils and exerts cytotoxicity by increasing membrane permeability or disturbing a cell’s lipid bilayer [36, 37].

In addition to eosinophils, we found an increase in the accumulation of macrophages in Th2 cells treated tumors. The presence of tumor-associated macrophages seems to be a crucial component of gastrointestinal cancers prognosis [38]. A study presented by Lorvik et al. noted that immune responses mediated by Th2 cells are responsible for M2 macrophage infiltration into myeloma where they produce arginase, and adoptive transfer of Th2 cells combined with blockade of arginase led to improvement of myeloma [21]. Our results show that Th2 cells favour macrophages accumulation, but also seem to be responsible for macrophages activation. In fact, in gastrointestinal tumors treated with Th2 cells, we noted enhanced production of MPO and NOS2, which has cytotoxic effects on colon and pancreas cancer cells as we have previously noted [39]. To note, MPO is produced by both neutrophils and macrophages; nevertheless we did not observe an increase of neutrophils in our models. Th2 cells in TME of gastrointestinal cancers affected not only phenotype and activity of macrophages, but also seem to be responsible for eosinophil activation.

In our study, we also observed large expression of cytotoxic granules and apoptotic factors in tumors treated with Th2 cells. While the cytotoxic action of CD8^+^ T cells or Th1 cells on tumor cells is well characterized, the significance of Th2 cells or eosinophils as effector cells in tumor cell death machinery is unclear [40]. Our in vitro results obtained from tumor-killing assay indicated that both Th2 cells and eosinophils are able to kill pancreas cancer cells mediated through caspase processing, which highlighted an essential role of both types of immune cells in the promotion of anti-tumorigenic activity. According to our findings, eosinophils are more prone to kill pancreas cancer cells than Th2 cells; however, it should be also mentioned that this is the first evidence that Th2 cells are characterized by cytotoxic ability against cancer cells.

Finally, using multiplex analysis, we find that IL-5, but not IL-4 or IL-13 is hypersecreted from colon and pancreas tumors obtained from mice treated with Th2 cells. Surprisingly, some groups have shown that IL-4 induces tumor clearance by promoting granulocytes infiltration [28]. However, we detected only a low level of IL-4 production in our studies. Moreover, using recombinant IL-5 and immunodeficient mice with BRAF and P5K1940 cell-derived tumors, we were able to show that IL-5 suppressed progression of both gastrointestinal cancers. Our findings are in line with a previous data provided by Simson et al. who noted that overexpression of IL-5 prevented establishment and growth of fibrosarcoma [8]. Ikutani et al. demonstrated that mice with defective IL-5 signalling or treated with antibodies against IL-5 are characterized by enhanced lung melanoma metastasis and impaired eosinophil regulation [9], suggesting that the impact of IL-5 in cancer needs to be further examined. An association between IL-5 and eosinophils was observed in melanoma where IL-5 administration was related to enhanced eosinophils infiltration and anti-metastatic activity in the lung [9]. On the other hand, one experimental report has suggested that IL-5 may have some tumor-promoting properties, but this was in mice with IL-5 depletion, which would also have diminished eosinophils activity [41]. The above-mentioned line of evidence highlighted that IL-5 acts as a potent anti-tumorigenic factor, which may affect action of eosinophils; however, the significance of Th2 cells in this phenomenon was not reported. We showed an association between the reduction of tumor size and enhanced eosinophils influx into gastrointestinal tumors in association with IL-5 after Th2 cells administration. This also occurred in mice where recombinant IL-5 treatment was employed. The differences of eosinophils influx between mice with gastrointestinal cancers treated with recombinant IL-5 and Th2 cells may be related with the innate anti-tumorigenic immune response amplified by directly recruiting immune cells into tumor and with more complex action of Th2 cells, which participate not only to eosinophils activation but also macrophage activity. In addition, subsequent studies found that eosinophils could directly kill BRAF and P5K1940 cancer cells by generation of cytotoxic and apoptotic factors. One study suggested that Th2 cells and eosinophil function may be supported by mast cells. The bidirectional cross-talk between mast cells and eosinophils was documented where MBP secreted from activated eosinophils may support mast cell function [42]. On the other hand, in addition to Th2 cells, mast cells are able to produce IL-5 and may be partially involved in anti-tumorigenic immune response and modulation of TME in gastrointestinal cancers [43]. However, the impact of mast cells on the innate anti-tumorigenic immune response mediated by Th2 cells should be verified in further studies.

From a treatment standpoint, there is potential of polarizing the tumor microenvironment toward type II immune responses. Currently, surgical intervention and therapy against gastrointestinal cancers such as chemotherapy and agents targeting surface receptors, angiogenesis, DNA damage response and cell cycle arrest or signalling pathways are available and validated in clinical trials [44]. Nevertheless, gastrointestinal cancer patients manifest therapy resistance and more effective treatments are needed to improve patient outcomes. Immunotherapies and therapies targeting tumor stroma as well as adoptive cell therapies seem to be promising with directed tumor-specific action and elimination of undesirable effects. There are numerous strategies affecting the modulation of function and action of macrophages and CD4^+^ or CD8^+^ T cells, which are being investigated progressively in clinical trials [45]. However, our study suggests the anti-tumorigenic action of Th2 cells, which is dependent not only on the direct action of Th2 cells on gastrointestinal cancer cells, but also on the reprogramming of the TME in gastrointestinal cancers, which could lead to some new therapy options. A therapy based on Th2 cells or IL-5 may be a valuable alternative for patients with gastrointestinal cancers and should be considered as a new treatment option for patients with colon and pancreas cancers. A better understanding of the role of Th2 cells and its ability to activate anti-tumorigenic immune response in gastrointestinal cancers may help improve further therapies and be utilized to develop novel combination therapy approaches.

## Conclusions

Together, these studies indicate that Type II immune responses prevent colon and pancreas tumor growth. Our results show that reprogramming of tumor microenvironment by Th2 cells leads to recruitment of macrophages and eosinophils. Functionally, Th2 cells affect macrophage polarization and activation of both macrophages and eosinophils, which leads to a significant anti-tumorigenic response by generating cytotoxic and apoptotic factors. Additionally, our data highlights the significance of IL-5 in the promotion of anti-tumorigenic activity mediated by Th2 cell and eosinophil cross-talk in gastrointestinal cancers. Overall, while it is common for cytokines and immune cells to have dual functions in cancer and other diseases, our data suggest that type II immune responses deserve more investigation for their potential to promote anti-tumorigenic immunity and their potential as therapeutic targets.

## Supplementary information

Supplementary Figure S1.

Checklist

## Data Availability

The datasets generated and/or analyzed during the current study are available from the corresponding author on reasonable request.
