# Supplementary material for: Th2 cells inhibit growth of colon and pancreas cancers by promoting anti-tumorigenic responses from macrophages and eosinophils
Source: Br J Cancer. 2022 Nov 14;128(2):387–97. doi: 10.1038/s41416-022-02056-2 (PMC9902541; doi:10.1038/s41416-022-02056-2)
Supplement: Supplementary file 1 — Supplementary Figure S1. [file 41416_2022_2056_MOESM1_ESM.pdf]

SUPPLEMENTARY INFORMATION

Th2 cells inhibit growth of colon and pancreas cancers promoting anti-tumorigenic responses by macrophages and eosinophils

Damian Jacenik[1,2], Ioannis Karagiannadis[2] & Ellen J. Beswick[2]

[1] – Department of Cytobiochemistry, Faculty of Biology and Environmental Protection, University of Lodz, Lodz, Poland.
[2] – Division of Gastroenterology, Department of Internal Medicine, University of Utah, Utah, Salt Lake City, United States.

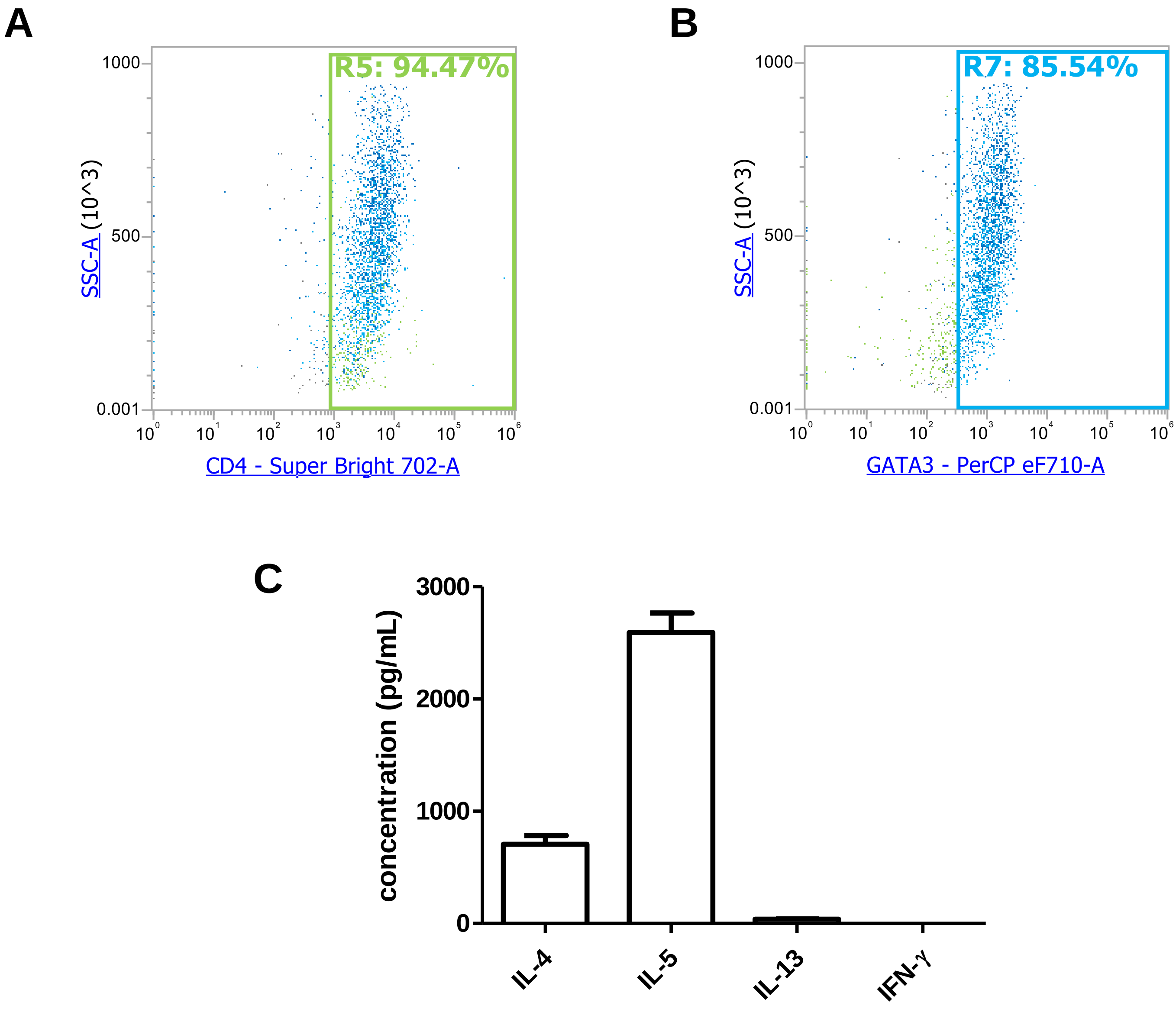

Supplementary Figure S1. Naïve T cells isolated from spleens of C57BL/6 wild type mice using negative selection and polarized toward Th2 cells show high levels of CD4 (A) and GATA3 (B) staining and produce high levels of IL-5 (C).
